# Neural stem cell-derived extracellular vesicles: mini players with key roles in neurogenesis, immunomodulation, neuroprotection and aging

**DOI:** 10.3389/fmolb.2023.1187263

**Published:** 2023-05-09

**Authors:** Valentina Bonetto, Mariagrazia Grilli

**Affiliations:** Department of Pharmaceutical Sciences, Laboratory of Neuroplasticity, University of Piemonte Orientale, Novara, Italy

**Keywords:** neural stem cells, extracellular vesicles, neurogenesis, hippocampal neurogenesis, neuroprotection, aging, immunomodulation, hypothalamus

## Abstract

Neural stem/progenitor cells (NSPCs) are self-renewing and multipotent cells of the central nervous system where they give rise to neurons, astrocytes and oligodendrocytes both during embryogenesis and throughout adulthood, although only in a few discrete niches. NSPC can integrate and send a plethora of signals not only within the local microenvironment but also at distance, including the systemic macroenvironment. Extracellular vesicles (EVs) are currently envisioned as main players in cell-cell communication in basic and translational neuroscience where they are emerging as an acellular alternative in regenerative medicine. At present NSPC-derived EVs represent a largely unexplored area compared to EVs from other neural sources and EVs from other stem cells, i.e., mesenchymal stem cells. On the other hand, available data suggest that NSPC-derived EVs can play key roles on neurodevelopmental and adult neurogenesis, and they are endowed with neuroprotective and immunomodulatory properties, and even endocrine functions. In this review we specifically highlight major neurogenic and “non-neurogenic” properties of NSPC-EVs, the current knowledge on their peculiar cargos and their potential translational value.

## 1 Introduction

Neural Stem cells (NSC) are self-renewing and multipotential cells that make vital contribution to CNS formation during development by generation of neurons, astrocytes, oligodendrocytes. When neurodevelopment is completed, NSCs stop proliferating in most CNS regions and undergo terminal differentiation or acquire a quiescent state. In the postnatal and adult periods, a few discrete CNS areas still retain active neurogenic niches, highly specialized microenvironments where NSC can generate adult born neurons. In mammalian brain adult NSCs reside in the subgranular zone (SGZ) of hippocampal dentate gyrus (DG) and in the subventricular zone (SVZ) (Eriksson et al., 1998; Li and Zhao, 2008). Recently hypothalamus and meninges have been described as regions where adult neurogenesis occurs (Nakagomi et al., 2011; Bifari et al., 2017; Sharif et al., 2021). In these specialized locations, adult NSCs transition from quiescent (qNSC) and activated stages (aNSC), and give rise to neural progenitor cells (NPC) which, in turn, can commit to differentiation towards neuronal and glial lineages. Adult born neurons eventually migrate to their final destination where they integrate into preexisting functional networks (Nakagomi et al., 2011).

Neural Stem/Progenitor Cells (NSPC) act not only autonomously but they integrate a plethora of signals communicated by local, distal, and systemic actors (Cvijetic et al., 2017; Spampinato et al., 2019). These different signals provided by macroenvironment and cells in the neurogenic niches modulate both NSCs and NPCs, by governing quiescence or activation transition, symmetric/asymmetric self-renewal, and fate specification decisions (Zhang et al., 2021).

Extensive work suggest that NSC may exert, in addition to neurogenic and regenerative functions, also “non-neurogenic” functions, due to their immunomodulatory and neuroprotective activities (Butti et al., 2014; Bacigaluppi et al., 2020). Such non-neurogenic functions mainly rely on the highly secretory nature of NSCs. They indeed release a vast array of molecules that act in an autocrine and paracrine manner in homeostatic and non-homeostatic conditions, suggesting a potential role, not only in intercellular communication and cell fate definition, but as effectors in additional brain and systemic functions and/or in CNS disorders (Paredes et al., 2018; Tata and Ruhrberg, 2018; Dause et al., 2022; Willis et al., 2022).

One of the main players in NSPC local and distant communication are extracellular vesicles (EVs), secreted membrane-delimited particles either originating from the endosomal system (exosomes, exo) or shed from plasma membrane (microvesicles). EVs content (nucleic acids, proteins, lipids and organelles) is involved in key cell-to-cell communication under physiological and pathological conditions (Tricarico et al., 2017; Tata and Ruhrberg, 2018; van Niel et al., 2018; Dause et al., 2022).

EV cargo sorting is significantly influenced by the environment and EVs derived from different sources may have unique properties relative to cell type; therefore, NSC-EVs content is highly sensitive to signals coming from other cells and can change in a strictly context-dependent manner (Cossetti et al., 2014). One major challenge for studying EVs is their standardized isolation and characterization. In addition to conventional methods like ultracentrifugation and ultrafiltration, novel techniques including microfluidic chips are under development (Akbar et al., 2022; Chen et al., 2022).

Results collected from different studies have contributed to the concept of NSPC-EVs as potential therapies for several CNS disorders, given their ability to pass the blood-brain barrier and their regenerative and immunomodulatory role (Ni et al., 2023). Despite such tremendous potential and expectations, available data on the NSPC-EVs are still underrepresented compared to studies on other neural cell types.

**FIGURE 1:**
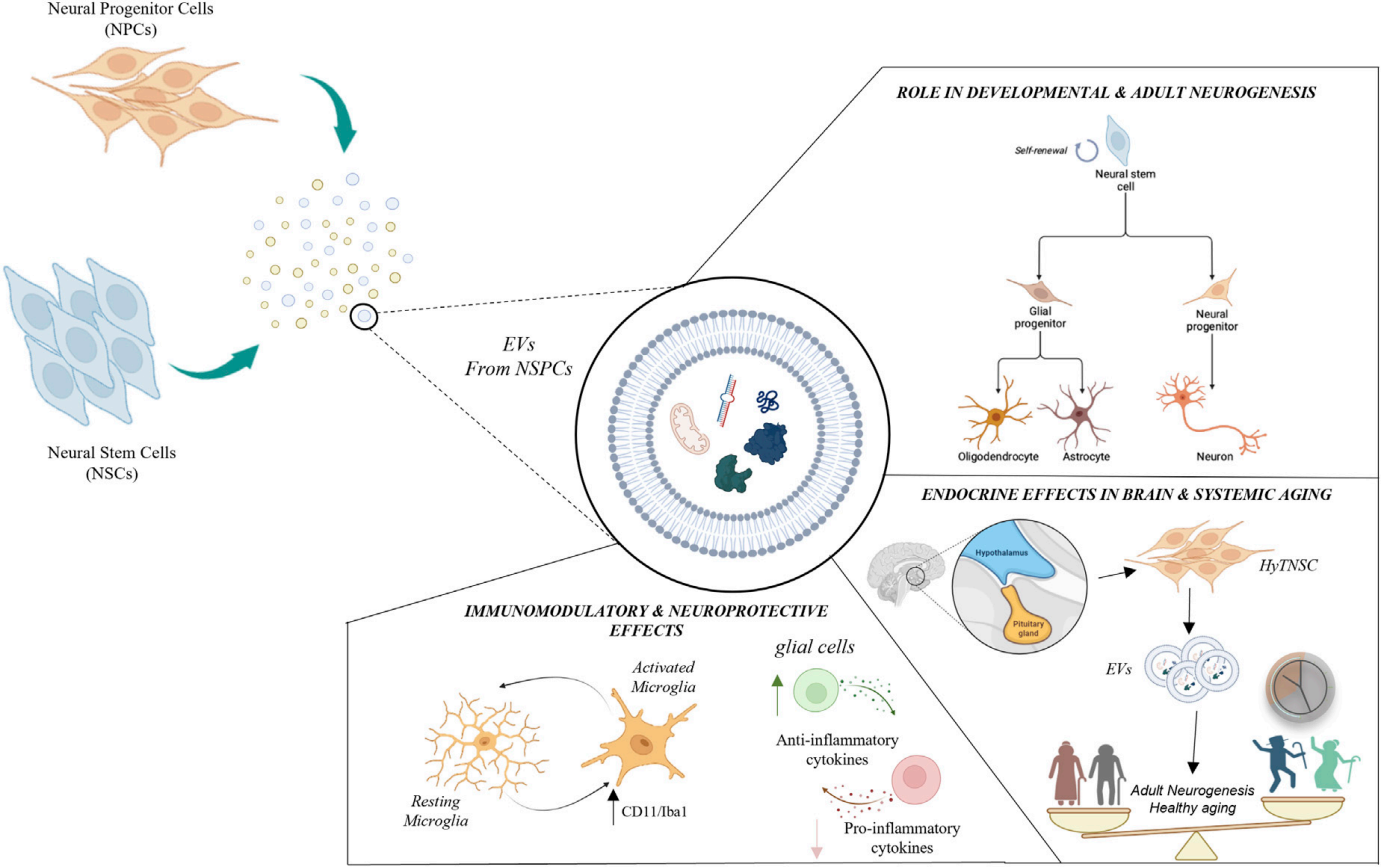
Schematic representation of major roles played by neural stem/progenitor cells-derived extracellular vesicles (NSPC-EVs). NSPC-EVs contain nucleic acids (e.g., DNAs, mRNAs, miRNAs, lncRNAs, etc.), proteins, lipids and organelles which may produce different biological functions in recipient cells. NSPC-EVs may exert important autocrine and paracrine functions influencing neuro- and glio-genesis process, both during brain development and in adult neurogenic niches. Conversely, NSPC-EVs may also play relevant “non neurogenic” functions, including immunomodulatory, neuroprotective and endocrine activities. EVs have been found to regulate immunity and protect neurons through modulation of microglia activation (characterized by the expression of membrane markers as CD11b and Iba1b) and anti-inflammatory effects. EVs derived from hypothalamic NSC (HytNSC) play key endocrine properties, both on brain and systemic ageing.

In this minireview we will summarize the current knowledge on EVs derived from NSPCs, discussing their potential role in the modulation of relevant physiological and physiopathological cellular processes. Specifically, we will focus on NSPC-EVs as carriers of active biomolecules with key roles in neurodevelopmental and adult neurogenesis, neuroprotective, immunomodulatory and endocrine functions, as represented in Figure 1.

## 2 NSPC-derived EVs: role in developmental and adult neurogenesis

EVs derived from NSCs may exert important autocrine and paracrine functions in neurogenesis, both during brain development (Sharma et al., 2013) and in adult neurogenic niches (Losurdo and Grilli, 2020). The majority of available literature data derive from *in vitro* studies, although a few studies also report the effect of EV administrations in animal models. Cultured NSPCs produce and secrete EVs both under proliferating and differentiating conditions and may affect any aspect of the neurogenesis process. EVs derived from differentiated mouse embryonal NSPCs were reported to elicit differentiation of proliferating NSPC (Stronati et al., 2019). Proteomic studies of EVs secreted during quiescence and proliferation NSC stages, together with use of an inhibitor of EV secretion, confirmed that they are involved both in quiescence maintenance and in NSC transition between proliferation and quiescence (Zhang et al., 2021). The study also suggested that qNSCs may use EVs to discard selected proteins, including ribosomal proteins (enriched in qNSC-derived EVs), to control quiescence and maintain low protein translation. Interestingly, PCSK6 (Anacker and Hen, 2017), a convertase that promotes cell proliferation via ERK1/2 and STAT3 pathways was enriched in aNSC-EVs (Zhang et al., 2021). Overall these interesting findings may help to identify, among aNSC-EVs associated proteins, potential candidates to reactivate NSCs in and counteract age- or pathology related dysfunction of NSC in neurogenic niches.

Insight into the complex actions of NSC-EV cargos has been provided by next-generation sequencing of RNAs in human NPC-EVs (hNPC-EVs). These efforts identified specific exosomal miRNAs that are differently expressed compared to the parent cell, with roles in neural regeneration, aging and neural plasticity. Stevanato et al. (2016) characterized a unique set of miRNAs enriched in exosomal versus cellular preparations from a clinical grade human NSC (hNSC) clonal cell line. They found a number of differentially expressed miRNAs in hNSCs compared with hNSCs-exos, with hsa-miR-1246, hsa-miR-4488, hsa-miR-4508, hsa-miR-4492 and hsa-miR-4516 being the most enriched miRNAs in exosomes (Stevanato et al., 2016). Careful stoichiometric analysis allowed to propose that these miRNAs may have significant effects on target cells in neurogenic niches. Moreover miR-9, highly expressed in primary embryonic NSCs-exos, triggered *in vitro* differentiation and maturation of NSCs toward both neuronal and glial lineages via Hes-1 modulation (Yuan et al., 2021).

Potentially relevant information also derived by comparison between primary NPC-EVs and EVs produced by NPC obtained by direct conversion of somatic cells (induced NPC, iNPC). *In vitro* experiments showed that, unlike primary NPC-EVs, iNPC-EVs had no proneurogenic effects (Ma et al., 2021). miR-21a was identified as highly enriched in primary cell-derived exosomes and having a key role in the generation of newborn neurons, via targeting key regulators of NPC proliferation and differentiation, such as Sox2 and Stat3. Similarly, miR-9 and miRlet-7b, which are upregulated and downregulated, respectively, in NPC-EVs compared to iNPC-EVs, suppressed expression of the orphan nuclear receptor TLX, known to maintain adult NSCs in an undifferentiated, proliferative state (Shi et al., 2004). In addition to miRNAs, EV-associated growth factors were correlated with cell-to-cell communication properties of NSCs, as observed in iNPCs derived from mouse astrocytes (Shi et al., 2004).

Emerging data suggested that EVs from different cell types are able to propagate specific metabolic signals to surrounding cells (Sano et al., 2014). By untargeted and targeted metabolomic analyses, Iraci and others (Iraci et al., 2017) demonstrated that human and mouse NSC-derived EVs exhibit enzymatic activities affecting both release and consumption of metabolites in the extracellular space. In particular, high L-asparaginase-like protein 1 (Asrgl1) activity is transferred by adult NSC to EVs, resulting in depletion of extracellular asparagine and release of aspartate, a metabolite with a role in cellular bioenergetics, essential for cells with impaired mitochondrial functions (Birsoy et al., 2015; Sullivan et al., 2015). Also other metabolites with key biological functions were substantially altered by NSC-EVs (GABA, Glutamate, lactate and alanine). Altogether these findings support the concept that, through their EVs, NSC may profoundly affect metabolic signals and, in turn, functional properties of surrounding cells.

Despite NSC-derived EVs *in vivo* studies are very limited in number, in a few studies EVs isolated from NSC derived from iPSC and injected into rodents confirmed their role in neurodevelopment (Cossetti et al., 2014) and in regulation of adult hippocampal neurogenesis (Sullivan et al., 2015; Seguro et al., 2018; Gharbi et al., 2023). In particular, intranasal administration of EVs from human iPSC-derived NSCs into adult rodents demonstrated that they could target and get incorporated into neurons and glia in almost all brain regions, including the DG (Gharbi et al., 2023). EV-treated rats displayed a higher density of Ki-67^+^and DCX^+^/BrdU^+^ cells in the SGZ suggesting that their cargo increases proliferation in the adult neurogenic niche (Gharbi et al., 2023). Moreover, hiPSCs-NSCs derived EVs were enriched in proteins such as Agrin and pentraxin-3 previously shown to play a role in adult hippocampal neurogenesis (Rodriguez-Grande et al., 2015; Zhang et al., 2020).

NSC-EV cargos may also change in response to local and systemic signals. Hypoxic preconditioning of NSC elicits significantly increased EVs release *in vitro* and resistance to hypoxia. Proteomic analysis of EVs from hypoxic and control NSCs demonstrated significant changes in EVs protein cargo in response to hypoxia and identified several candidate proteins that play a pivotal role in the cell-to-cell mediated communication underlying NSC and progeny survival and neuronal differentiation (Birsoy et al., 2015).

In response to the neurotoxin kainic acid, anti-apoptotic miR-124 and miR-137 are upregulated in primary cultures of adult hippocampal NSCs (ahNSCs). Remarkably, NSCs selectively sort only miR-124, and not miR-137, into their secreted exosomes (Upadhya et al., 2020). This molecular sorting may reflect in part how NSCs can decide, via EVs, to rewire the hippocampal circuit network by promoting maturation and survival of newly born DG cells.

NSC-EV cargo has been demonstrated to be sensitive to viral infections. ZIKA virus (ZIKV) infection in pregnant women produces dramatic neurodevelopmental damage with microcephaly in children. A variety of host miRNAs which are incorporated in induced pluripotent stem cell (iPSC)-derived hNSC-EVs infected with ZIKV were identified. Interestingly, differentially incorporated miRNAs in EVs were associated with processes related to neural and embryonic development, including axon guidance and FoxO signaling (Gharbi et al., 2023). Similar studies are relevant since they provide key information about pathogenic mechanisms underlying severe neurodevelopmental alterations and contribute to a better understanding of the role of NSC-EVs in normal brain development.

## 3 NSPC-derived EVs: immunomodulatory and neuroprotective properties

In adult brain NSPCs reside in dynamic microenvironments within proximity to the systemic circulation and the cerebrospinal fluid. Therefore, they do respond to factors not only present locally, but also present in other brain regions or in the systemic macroenvironment. Increasing evidence proposed that EVs confer immunomodulatory and anti-inflammatory effects in various inflammatory disorders and tissue injury by suppressing activation of immune cells and reducing pro-inflammatory mediators production (Akbar et al., 2022; Li et al., 2022). EVs have been found to regulate immunity and restore function through a variety of pathways. As an example, inhibition of MAPK, an inflammatory related pathway, can be triggered by different Let-7 family miRNAs in EVs (Morton et al., 2018; Tian et al., 2021). These miRNAs act directly on downstream gene targets of STAT3 and immunosuppressive proteins, such as PD-L1 and arginase-1, while indirectly, they interfere with expression and processing of major histocompatibility complex (MHC) class II molecules in antigen-presenting cells (Bian et al., 2020; Adamus et al., 2021).

### 3.1 NSPC-derived EVs: effects on resident brain immune cells and inflammation

Microglia are the most abundant resident immune cells in the CNS and are involved in different physiological processes, including synaptic pruning and remodeling, phagocytosis and elimination of CNS-threatening antigens (Xu et al., 2022). They also reside in neurogenic niches where they play a regulatory role under physiological and pathological conditions (Xu et al., 2022).

NSPC-EVs can contribute to regeneration and maintenance of an anti-inflammatory state through immunoregulatory mechanisms. NPC-EVs intrinsic immunomodulatory activity was demonstrated both *in vivo* and *in vitro*, with significant suppression of microglia activation, prevention of reactive astrogliosis, and attenuation of inflammation-induced neurodegeneration (Morton et al., 2018). NSC-EVs associated miRNAs were proposed as main drivers of phenotypic changes in recipient glial cells within neurogenic niches. Previous studies demonstrated the presence of highly enriched NSC-EV miRNAs, namely, miR-9, miR-let-7, miR-26a, and miR-181c, with key roles in regulating microglia morphology and physiology (Åkerblom et al., 2013; Zhang et al., 2015; Guo et al., 2019). Exposure to NSC-EVs produces changes in microglial transcriptional state with Let-7-regulated cytokine release causing a negative feedback loop on NSC proliferation (Morton et al., 2018; Guo et al., 2019). Similarly, *in vivo* injection of conditioned media derived from NPC-EV-treated microglia into the lateral ventricles reduced SVZ NPCs proliferation, again suggesting a NPC-microglia cross-talk that ultimately generates a negative feedback loop onto NPCs (Morton et al., 2018). These findings are also supported by the observation that *in vitro* exposure to NSC conditioned medium (NSC-CM), containing exosomes, induces a significant reduction of TNF-α/IL-6 release and Iba1 expression in primary microglia (Morton et al., 2018; Tian et al., 2021).

Additional studies suggested that NSC-EVs play an important role in the modulation of inflammatory responses in conditions of CNS damage. In a mouse model of traumatic spinal cord injury NSC-EVs morphogen activity on microglia coupled with a decrease in the amount of nitric oxide (NO) produced by macrophages and reduced levels of pro-inflammatory cytokines. These effects could be mediated by EV-associated 14–3-3t, a protein which modulates beclin-1 expression, reduces apoptosis and neuroinflammation, and activates autophagy (Rong et al., 2019a; Rong et al., 2019b).

Cossetti et al. (Cossetti et al., 2014) demonstrated that pro-inflammatory (Th1-like) stimulation of NPC causes the release of NPC-EV-bound IFN-γ capable of activating signaling pathways in recipient cells. Given the largely documented evidence of IFN-γ signaling in both microglia and astrocytes, these findings suggest that under inflammatory conditions NSC-derived EVs preferentially or selectively target those glial phenotypes.

### 3.2 NSPC-derived EVs: neuroprotective properties

NSC-derived EVs have been shown to exert neuroprotective effects both *in vitro* and *in vivo*.

Micci et al. (Micci et al., 2019) isolated exosomes from hippocampal murine NSC and investigated their ability to promote synaptic resilience to amyloid-β (Aβ) oligomer-mediated toxicity. When administered ICV to mice, NSC-exos abolished Aβ-induced suppression of LTP and subsequent memory deficits. Once again, a set of NSC-exo enriched miRNAs was demonstrated to mediate *in vivo* beneficial effects (Micci et al., 2019).

Excessive ROS-induced mitochondrial dysfunction is an established and common feature of a wide range of neurodegenerative and inflammatory CNS disorders. EVs from F3 cells, a human fetal immortalized NSC line, attenuated oxidative stress and protected dopaminergic neurons from degeneration induced by 6-hydroxydopamine *in vitro* and *in vivo* (Micci et al., 2019). EVs derived from F3 and hNSPC enhanced the expression of antioxidant enzymes like superoxide dismutase through the modulation of PI3K/Akt and ERK-related signaling pathways, resulting in ROS scavenging and cell survival (Liu et al., 2020; Ma et al., 2021). EVs secreted by rat NSCs subjected to heat shock (HS) exhibited greater neuroprotective *in vitro* activity against oxidative stress and Aβ neurotoxicity, compared to EVs from control NSCs. Mass spectrometry studies and gene ontology (GO) enrichment analysis suggested that the top biological functions for proteins in HS-NSC-EVs are negative regulation of apoptosis and positive modulation of DNA repair (Huber et al., 2022). NSC-EVs were also shown to promote cell survival by enhancing autophagy, which reduces excessive toxic substances in both pathological and physiological conditions (Rong et al., 2019b).

Interestingly, NSC-derived EV cargo may include organelles. Untargeted proteomics revealed an enrichment of mitochondrial proteins in NSC-derived EVs (Peruzzotti-Jametti et al., 2021). Ultrastructurally intact and functionally preserved mitochondria are in NSC-EVs and could be transferred to restore mitochondrial function in target cells both *in vitro* and *in vivo* in a rodent model of multiple sclerosis (MS). Amelioration of MS clinical scores in mice provided the first evidence that EVs may represent a future acellular approach aimed at restoring mitochondrial dysfunction in neurological disorders (Peruzzotti-Jametti et al., 2021).

A few studies not only provided demonstration of NSC-EV-mediated neuroprotective effects but proved their uptake by target cells. In a rat model of retinal degeneration (RD), one of the most frequent causes of visual impairment and blindness, direct administration of mouse NPC-derived exosomes (mNPC-exos) delayed photoreceptor degeneration and apoptosis and preserved visual function (Bian et al., 2020). Authors demonstrated that mNPC-exos were specifically internalized by retinal microglia and suppressed their activation through a set of specific miRNAs. Such miRNAs markedly inhibited inflammatory signal pathways by targeting TNF-α, IL-1β, and COX-2 mediated inflammatory pathways. Interestingly, grafted human NPCs secrete EVs in the subretinal space of RD mice. Also hNPC-exos contained miRNAs that inhibited microglial activation, opening to potential translational applications of such findings.

## 4 NSC-derived EVs: endocrine effects in brain and systemic aging

EVs derived from endogenous NSC exert important effects not only locally, but even at distance, including systemically. A very interesting example of this concept resides in studies focusing on hypothalamic neural stem cells (HytNSC), which are responsible for adult neurogenesis occurring in this region. Currently, hypothalamic neurogenesis is believed to be implicated in the control of energy metabolism, reproduction and sleep (Sharif et al., 2021). A few years ago the physiological relevance of adult hypothalamic neurogenesis in systemic aging has been suggested (Zhang et al., 2013). Later, the same research group demonstrated a remarkable loss of HytNSC in middle-aged mice and that in rodents *in vivo* ablation or replenishment by transplantation of these cells can result in acceleration and slowdown of aging, respectively (Zhang et al., 2017). Of note, the authors also demonstrated that HytNSC-exos not only modulated the local neurogenic niche but also contributed with their cargo to a pool of CSF miRNAs. During aging specific miRNAs appeared downregulated in secreted exosomes. When EVs of postnatal cultured HytNSC were injected in the third ventricle of middle-aged mice, they reversed age-related physiological decline, and in particular improved locomotion, coordination, memory performance and sociality, in comparison to vehicle-treated mice. Also transplantation of healthy hypothalamic NSCs into the aging brain maintained the concentration of miRNAs at optimal levels through EV release, with successful aging and lifespan extension (Zhang et al., 2017). Altogether, these exciting data underlie the relevance of an endocrine function of HytNSCs which, mainly through EV-miRNAs, can exert control on both brain and systemic aging speed. Although at present speculative, the implications of these studies are relevant for future anti-aging strategies.

## 5 Conclusion and perspectives

The field of extracellular vesicles represents a fast progressing area in basic and translational neuroscience where EVs have emerged as a potential alternative in regenerative medicine. EVs can be secreted by any given cells in the CNS and are hypothesized to play a critical role in cell–cell communication over short range, but also widely within brain through the cerebrospinal fluid and with the periphery.

In this review we have specifically focused on summarizing the current knowledge on some peculiar properties of NSPC-derived EVs as reported in Table 1, a relatively unexplored area of research when compared to much larger evidence on the biological roles of EVs from neurons and glial cells. According to a consolidated view, NSCs have the ability to generate functional neural cell types, including neurons, but they may also provide relevant protective and reparative functions in CNS through their “non-neurogenic” properties. Their secretome, including EVs, likely possesses very similar properties which need to be better exploited for future translational purposes. This field of experimental activities is at an initial stage. At present most studies on the repertoire of NSPC EV-associated cargos have been mainly focused on miRNAs but investigation on other biological molecules, including lipids or even intact organelles associated with EVs, are highly required for a more comprehensive view of their biological significance. Also more studies comparing EVs generated from NSPCs located in distinct neurogenic niches are needed. These efforts may unravel novel candidate biological molecules that may provide neurogenic and non-neurogenic activities on specific cell phenotypes and/or in a spatially restricted manner.

**TABLE 1:** List of different effector molecules and organelles identified in extracellular vesicles derived from neural stem/progenitor cells-(NSPC-EVs) based on their role in neurodevelopment and neurogenesis, their immunomodulant and neuroprotective activities, and, finally, in brain and systemic ageing.

Function	Class of molecules/Organelle	Molecules	Outcome/mechanism
Neurodevelopment and neurogenesis	Enzymes	Asrgl1	↑ levels of aspartate/glutamate (Iraci et al., 2017)
		PCSK6	↑ proliferation via ERK1/2 and STAT3 pathways (Zhang et al., 2021)
	miRNA	miR-21a	↑ proliferation and differentiation targeting Sox2 and Stat3 (Shi et al., 2004)
		miR-9	Modulation of Hes-1 triggering differentiation and maturation of NSCs (Shi et al., 2004)
		miR-9 and miR-let-7	↓ NSC proliferation and ↑ neural differentiation via TLX (Shi et al., 2004)
		miR-124	↑ NSC neuronal differentiation, fate specification and differentiation in SVZ modulating BCL2L3 (Gharbi et al., 2023)
	Growth factor	VEGF	↑ NSC proliferation in SGZ (Tabari et al., 2020)
	Protein	Agrin	↑Hippocampal neurogenesis and synaptogenesis (Rodriguez-Grande et al., 2015; Tabari et al., 2020)
		Pentraxin-3	Restored SVZ neurogenesis post MCAo *in vivo*; ↑angiogenesis via VEGF upregulation *in vitro* (Tabari et al., 2020)
Immunomodulatory activities	miRNA	Let-7 family	microglia activation that negatively affects NSC proliferation in SVZ (Åkerblom et al., 2013)
		miR-9, miR-let-7, miR-26a, and miR-181c	Morphogen activity on microglia (Åkerblom et al., 2013; Zhang et al., 2015; Xu et al., 2022)
	Cytokine	INFγ/IL-6	Regulate function of microglia and astrocytes via Stat1 in target cells (Li et al., 2022)
	Protein	14-3-3	Modulation of beclin1
			↓ apoptosis and neuroinflammation ↑authophagy (Guo et al., 2019)
Neuroprotection	Enzyme	Antioxidant (e.g., SOD)	↑ cell survival
			↑ ROS scavenging (Lee et al., 2022) via PI3K/Akt and ERK pathways (Ma et al., 2021)
	miRNA	miR-181, miR-26, miR-9 and Let-7 family	↓TNF-α, IL-1β and COX-2 signalling pathways with anti-inflammatory effects (Tian et al., 2021)
	Protein	Heat-shock protein (e.g., Hapa1a)	↓oxidative stress and Aβ neurotoxicity *in vitro* (Rong et al., 2019b)
	Organelle	Mitochondria	immunoregulatory effect and modulation of energetic metabolism *in vitro* and *in vivo* (Peruzzotti-Jametti et al., 2021)
Aging	miRNAs	e.g., miR-335-3p, miR-192	↓NF-κB activation, restored GnRH secretion in hypothalamus and control of ageing speed (Zhang et al., 2013)

## Abbreviations

NSPC, neural stem/progenitor cell; NSC, neural stem cell; NPC, neural progenitor cell; ahNSC, adult hippocampal neural stem cell; HytNSC, hypothalamic neural stem cell; qNSC, quiescent neural stem cell; aNSC, activated neural stem cell; iNPC, induced neural progenitor cell; hNSC/NPC/NPSC, human neural stem/progenitor cell; mNSC/NPC/NPSC, mouse neural stem/progenitor cell; iPSC, induced pluripotent stem cell; NSC-CM, neural stem cell conditioned medium; CNS, central nervous system; SGZ, subgranular zone; SVZ, subventricular zone; DG, dentate gyrus; EV, extracellular vesicle; exo, exosome.
