# Correction: Neural stem cell-derived extracellular vesicles: mini players with key roles in neurogenesis, immunomodulation, neuroprotection and aging

**DOI:** 10.3389/fmolb.2025.1667664

**Published:** 2025-07-25

**Authors:** Valentina Bonetto, Mariagrazia Grilli

**Affiliations:** Department of Pharmaceutical Sciences, Laboratory of Neuroplasticity, University of Piemonte Orientale, Novara, Italy

**Keywords:** neural stem cells, extracellular vesicles, neurogenesis, hippocampal neurogenesis, neuroprotection, aging, immunomodulation, hypothalamus

In the published article, the funder [MIUR Progetti di Rilevante Interesse Nazionale (PRIN)], [Grant 20202THZAW_005] to [MG] was erroneously omitted. The corrected funding statement appears below.

“This work was supported by MIUR Progetti di Ricerca di Rilevante Interesse Nazionale (PRIN) Bando 2017–grant 2017XZ7A37 and Grant 20202THZAW_005 to MG.”

The original article has been updated.

